# Sharper insight: diagnostic superiority of ultrasound in lateral elbow injuries

**DOI:** 10.12701/jyms.2026.43.48

**Published:** 2026-07-15

**Authors:** Hilmi Berkan Abacıoğlu, Ahmet Furkan Çolak, Berkay Yalçınkaya, Levent Özçakar

**Affiliations:** Department of Physical and Rehabilitation Medicine, Hacettepe University Medical School, Ankara, Türkiye

A 29-year-old man presented with pain, swelling, and restricted mobility of the left elbow that persisted for 10 days following trauma sustained during weight training. The injury most likely resulted from a sudden varus moment associated with an axial compression of the radiocapitellar joint during a heavy biceps curl performed with the forearm in supination. The patient reported progressive reduction in swelling and complete resolution of the initial redness (possibly attributable to a local hematoma); however, the pain and functional limitations persisted. The initial plain radiographs obtained in the emergency department showed no pathological findings. Computed tomography (CT) performed during the same emergency department visit was normal.

Physical examination revealed painful and mildly restricted flexion and extension of the left elbow. Forceful varus stress reproduced the lateral elbow pain without any evidence of mechanical instability or excessive joint laxity. Ultrasound examination revealed an avulsion fracture involving the capitellum/lateral epicondyle at the radial collateral ligament (RCL) footprint, together with common extensor tendinopathy, suggestive of concomitant lateral elbow complex (LEC) injury (Fig. 1A). Additionally, a small joint effusion was observed within the radial and coronoid fossae, consistent with the close anatomical relationship between the RCL complex and joint capsule, as the annular ligament (AL) is histologically continuous with the elbow joint capsule [1] (Fig. 1B, 1C). Dynamic ultrasound evaluation further demonstrated a radiohumeral synovial plica injury (Supplementary Video 1). Subsequent magnetic resonance imaging (MRI) confirmed mild-to-moderate joint effusion and bone marrow edema involving the coronoid process, capitellum, and lateral epicondyle, with questionable ligamentous injury (Fig. 2). In the absence of mechanical joint instability, and considering the patient’s symptomatic improvement, conservative management was initiated with a resting splint and oral diclofenac (75 mg twice daily). At the 3-week follow-up, ultrasonography demonstrated near-complete resolution of the effusion, whereas the avulsion fracture fragment remained visible. The splint was removed, and the patient was enrolled in a structured physical therapy program that included range of motion, stretching, and strengthening exercises. The patient’s subsequent follow-up was uneventful.

The LEC, which comprises the common extensor tendon (CET), RCL, lateral ulnar collateral ligament (LUCL), and AL, plays a central role in elbow stability. Underneath the CET, the RCL complex forms the deep ligamentous layer of the lateral elbow. It consists of the RCL, AL, and LUCL, which form a histological continuum. The RCL inserts into a bony footprint located deep and distal to the CET attachment on the lateral epicondyle. The AL is also histologically continuous with the elbow joint capsule, highlighting the intimate anatomical relationship between the lateral ligamentous complex and the capsule [1].

Avulsion fractures secondary to LEC injury are a well-recognized condition of elbow trauma. Given that LEC injuries are frequently associated with radiohumeral joint involvement, dynamic ultrasound evaluation of the radiohumeral synovial plica is clinically relevant [2]. Although MRI remains the most widely used modality for the assessment of elbow trauma in many clinical settings, ultrasonography has gained increasing acceptance owing to several advantages, including bedside availability, absence of ionizing radiation, real-time dynamic assessment, and the ability to incorporate advanced techniques such as sonopalpation and the sono-Tinel sign [3]. In the present case, ultrasonography was selected as the initial imaging modality. The combination of static and dynamic ultrasound examinations allowed for a more comprehensive characterization of the underlying pathology. However, the literature does not yet provide a single well-defined quantitative ultrasound criterion (e.g., a specific cutoff thickness) for radiohumeral synovial plica lesions. In our patient, the diagnosis was established based on increased intra-articular fluid and dynamic evidence of plical displacement on dynamic imaging. Although MRI was subsequently performed because of its established role in the evaluation of joint trauma and recognized utility in detecting cartilaginous injuries, it failed to identify the avulsion fracture and did not yield additional diagnostic or therapeutic benefits beyond those obtained with ultrasonography. Interestingly, we were unable to detect the avulsion fracture using CT in our patient. Similar missed diagnoses have previously been reported for small avulsion fractures, which subsequently became apparent on MRI owing to visualization of the surrounding soft-tissue edema [4]. However, in our case, we believe that the failure to detect the avulsion fracture on CT was related to technical factors, particularly the angular orientation of the fracture fragment. Considering the paucity of literature addressing the ultrasound evaluation of avulsion fractures associated with LEC injuries, this case underscores the potential diagnostic superiority of ultrasonography over other imaging modalities in the assessment of ligamentous injuries around the elbow [5].

## Figures and Tables

**Fig. 1.**
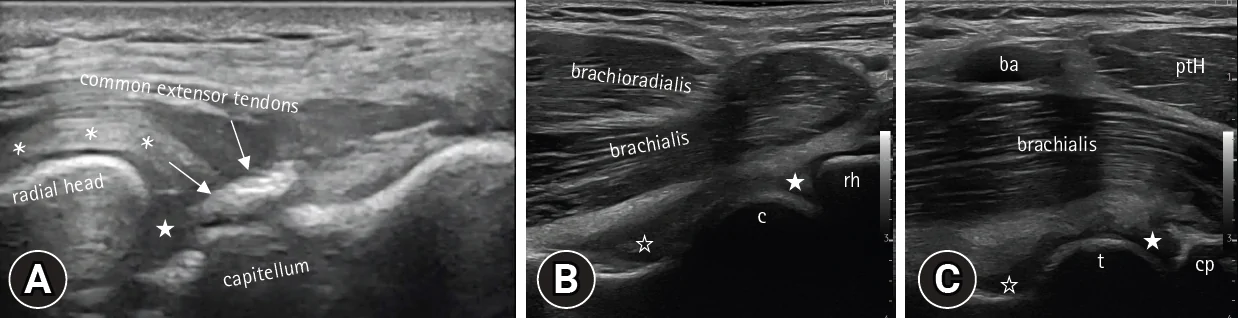
Sonographic examination of the symptomatic elbow reveals (A) an avulsion fracture (arrows), and (B, C) effusion (hollow stars) in the radial and coronoid fossae, respectively. Asterisks, radial collateral ligament; stars, radiohumeral synovial plica; c, capitellum; rh, radial head; ptH, humeral head of the pronator teres; t, trochlea; cp, coronoid process; ba, brachial artery.

**Fig. 2.**
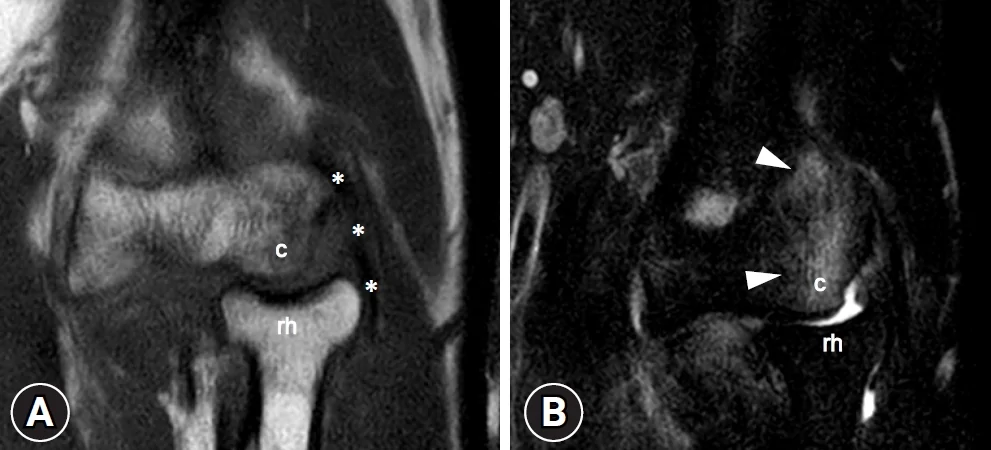
Magnetic resonance imaging demonstrates (A) an intact radial collateral ligament (asterisks) in T1-weighted sequence, and (B) bone marrow edema (arrowheads) of the capitellum in T2-weighted sequence. c, capitellum; rh, radial head.

## Supplementary materials

Supplementary Video 1 can be found at https://doi.org/10.12701/jyms.2026.43.48.

Supplementary Video 1. Dynamic real-time imaging demonstrates the avulsed bone fragment (arrow) along with the compromised radiohumeral synovial plica (star) during repeat forearm pronation/supination.
